# Centrosome amplification and aneuploidy driven by the HIV-1-induced Vpr•VprBP•Plk4 complex in CD4^+^ T cells

**DOI:** 10.21203/rs.3.rs-2924123/v1

**Published:** 2023-08-22

**Authors:** Jung-Eun Park, Tae-Sung Kim, Yan Zeng, Christina M. Monnie, Muhammad S. Alam, Ming Zhou, Melissa Mikolaj, Frank Maldarelli, Kedar Narayan, Jinwoo Ahn, Jonathan D. Ashwell, Klaus Strebel, Kyung S. Lee

**Affiliations:** 1 Cancer Innovation Laboratory, Center for Cancer Research, National Cancer Institute, National Institutes of Health, Bethesda, MD, 20892, USA.; 2 Department of Structural Biology and Pittsburgh Center for HIV Protein Interactions, University of Pittsburgh School of Medicine, Biomedical Science Tower 3, RM 1055, 3501 Fifth Ave., Pittsburgh, PA, 15260, USA; 3 Laboratory of Immune Cell Biology, Center for Cancer Research, National Cancer Institute, National Institutes of Health, Bethesda, MD, 20892, USA.; 4 Protein Characterization Laboratory, Frederick National Laboratory for Cancer Research, Leidos Biomedical Research, Inc., Frederick, Maryland 21702, USA; 5 Center for Molecular Microscopy, Center for Cancer Research, National Cancer Institute, National Institutes of Health, Bethesda, MD 20892, USA; 6 Cancer Research Technology Program, Frederick National Laboratory for Cancer Research, Frederick, MD 21702, USA; 7 HIV Dynamics and Replication Program, NCI, NIH, Frederick, MD 21702, USA; 8 Laboratory of Molecular Microbiology, National Institute of Allergy and Infectious Diseases, NIH, Bethesda, Maryland, USA; 9 These authors contributed equally to this work.

## Abstract

HIV-1 infection elevates the risk of developing various cancers, including T-cell lymphoma. Whether HIV-1-encoded proteins directly contribute to oncogenesis remains unknown. We observed that approximately 1–5% of CD4^+^ T cells from the blood of people living with HIV-1 exhibit over-duplicated centrioles, suggesting that centrosome amplification underlies the development of HIV-1-associated cancers by driving aneuploidy. Through affinity purification, biochemical, and cell biology analyses, we discovered that Vpr, an accessory protein of HIV-1, hijacks the centriole duplication machinery and induces centrosome amplification and aneuploidy. Mechanistically, Vpr formed a cooperative ternary complex with an E3 ligase subunit, VprBP, and polo-like kinase 4 (Plk4). Unexpectedly, however, the complex enhanced Plk4’s functionality by promoting its relocalization to the procentriole assembly and induced centrosome amplification. Loss of either Vpr’s C-terminal 17 residues or VprBP acidic region, the two elements required for binding to Plk4 cryptic polo-box, abrogated Vpr’s capacity to induce all these events. Furthermore, HIV-1 WT, but not its Vpr mutant, induced multiple centrosomes and aneuploidy in primary CD4^+^ T cells. We propose that the Vpr•VprBP•Plk4 complex serves as a molecular link that connects HIV-1 infection to oncogenesis and that inhibiting the Vpr C-terminal motif may reduce the occurrence of HIV-1-associated cancers.

## Introduction

A large body of evidence suggests that people living with HIV-1 are at high risk of developing various comorbid diseases, including cancer ^1^. While the weakened immune system brought about by HIV-1 infection is generally blamed for the increased risk of developing these disorders, several studies suggest that integration of HIV-1 proviruses in oncogenes could promote cellular transformation and the development of T-cell lymphomas ^2–5^. Interestingly, centrosome amplification, which is prevalent among hematological malignancies ^6–9^, constitutes the major causal mechanism of chromosomal instability and aneuploidy ^10^. These observations raise the possibility that HIV-1-encoded proteins may promote oncogenesis by deregulating the centrosome duplication process.

As the main microtubule-organizing center in animal cells, the centrosome (composed of a pair of barrel-shaped centrioles and their surrounding pericentriolar material) is critically required for normal cell division and proliferation ^11,12^. Tight control of centrosome number is fundamentally required for proper bipolar spindle formation, an event critical for accurate chromosome segregation and the maintenance of genomic integrity. Studies with cultured cells show that HIV-1 Vpr (viral protein R), a multifunctional molecular adaptor ^13–15^, localizes to the centrosome and can induce centrosome overduplication (i.e., more than two centrosomes per cell) ^16,17^. The mechanism underlying how Vpr induces centrosome overduplication at the molecular level remains unknown. Notably, Vpr binds to various cellular proteins ^13^, including VprBP/RIP, a high-affinity target of HIV-1 Vpr ^18^ with promiscuous scaffold functions ^19^. (VprBP is also named DDB1-CUL4-Associated Factor 1 [DCAF], a substrate receptor for the CUL4-DDB1 ubiquitin ligase ^20,21^). Several studies demonstrate that VprBP functions as a substrate recognition subunit of E3 ubiquitin ligases ^19,22,23^ and that VprBP interaction with Vpr or the structurally related Vpx is vital to detect specific cellular targets for their proteasomal degradation ^24–27^. Recent studies show that, in addition to localizing to the nucleus, VprBP localizes to centrosomes and promotes the degradation of CP110 ^17,28^, a conserved centriolar protein required for centrosome duplication ^29,30^. Whether VprBP has an uncharacterized role other than serving as a component of E3 ubiquitin ligases has yet to be discovered.

Polo-like kinase 4 (Plk4) is a key regulator of centriole biogenesis ^31–33^, which occurs precisely once per cell cycle ^10^. Plk4 is recruited to the outmost region of a pericentriolar scaffold Cep152 in early G1 ^34–37^ through the interaction between its non-catalytic cryptic polo-box (CPB) domain and the N-terminal region of Cep152 ^37^. As the level of Plk4 rises in late G1, it undergoes *trans*-autophosphorylation-dependent ^38^ liquid–liquid phase separation (LLPS) ^39–42^ and dynamic relocalization from around Cep152 (i.e., ring-like state) to the procentriole assembly site (dot-like state) ^36,43^. Dysregulation of this process results in abnormal centrosome numbers and chromosome instability that could lead to aneuploidy, a cause of cancer development ^10,44–46^.

Here we showed that the CD4^+^ T cells purified from the blood of people living with HIV-1, but not healthy individuals, exhibit overduplicated centrosomes in approximately 1–5% of the population. Subsequent analyses suggested that centrosome amplification was driven by the ability of Vpr to form a cooperative complex with VprBP and Plk4 and induce Plk4-mediated centriole overduplication. At the molecular level, Vpr, which binds to the VprBP WD40 domain ^27,47^, also interacts with Plk4 CPB through its C-terminal tail (CT; residues 80–96). The C-terminal acidic region (AR) of VprBP also interacted with the CPB of Plk4, establishing three-way interactions to form the Vpr•VprBP•Plk4 complex. Consistent with these findings, deletion of either the Vpr CT or VprBP AR significantly diminished the level of Vpr-induced centrosome overduplication and aneuploidy in various CD4^+^ cells, including primary T cells prepared from peripheral blood mononuclear cells (PBMCs) of healthy human subjects. Given that a modest elevation of Plk4 level (<2-fold) is sufficient to induce various tumors in a mouse model ^48^, the data provided here suggest that the HIV-1-induced Vpr•VprBP•Plk4 complex can promote oncogenesis by bolstering Plk4-dependent centriole duplication.

## Results

### Centrosome amplification in CD4^+^ T cells from people living with HIV-1.

**Fig. 1.**
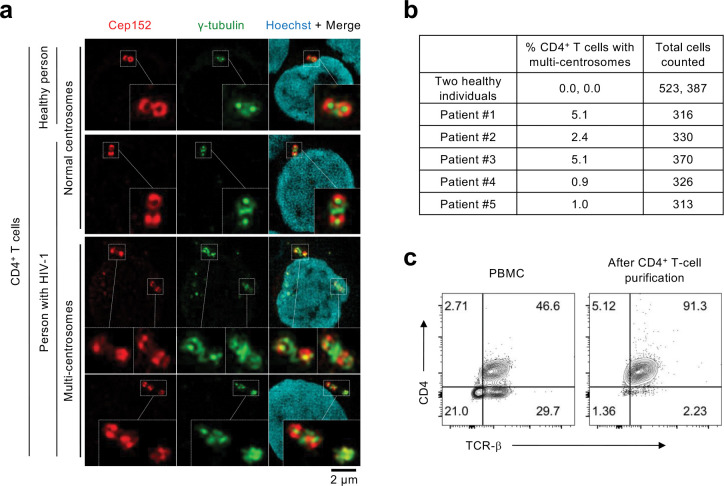
Centrosome amplification in CD4^+^ T cells purified from people living with HIV-1. **a,** Immunostained images showing over-duplicated centrosomes (marked by Cep152 and γ-tubulin signals) in primary CD4^+^ T cells purified from people living with HIV-1 but not healthy individuals. Boxes, area of enlargement. **b,** Quantified data showing the percentage of CD4^+^ T cells that exhibit multiple centrosomes. Quantification was performed with the cells purified from the blood of two healthy people and five people living with HIV-1 (clinical data are provided in Supplementary Fig. 1b). **c,** Flow cytometry data showing the percentage of CD4^+^ T cells before and after purification from PBMCs.

To explore whether HIV-1 can induce centrosome abnormalities, we performed immunostaining analyses with CD4^+^ T cells purified from the PBMCs of five individuals living with HIV-1 (before they developed HIV-1-associated disorders, including cancer). Doubly stained Cep152 and γ-tubulin signals were used as surrogate markers for a centrosome (Fig. 1a and Supplementary Fig. 1). To our surprise, although the degree of centrosome overduplication varied from 0.9% to 5.1% among different samples, the occurrence of cells with overduplicated centrosomes was manifest in all five cases examined (Fig. 1b). (Note the sample number is low because of the difficulty of obtaining HIV-1-positive PBMC cells.) Control CD4^+^ T cells from two healthy individuals showed no detectable level of overduplicated centrosomes. The purity of the CD4^+^ T cells was verified by the flow cytometry data shown in Fig. 1c. These findings suggest that HIV-1 infection may alter cellular processes that can lead to centrosome amplification and aneuploidy, a condition that can drive oncogenesis ^45,46^. Furthermore, since Plk4 is a master regulator of centrosome duplication, its function could have been deregulated under HIV-1 infection.

### VprBP AR interacts with Plk4 CPB.

**Fig. 2.**
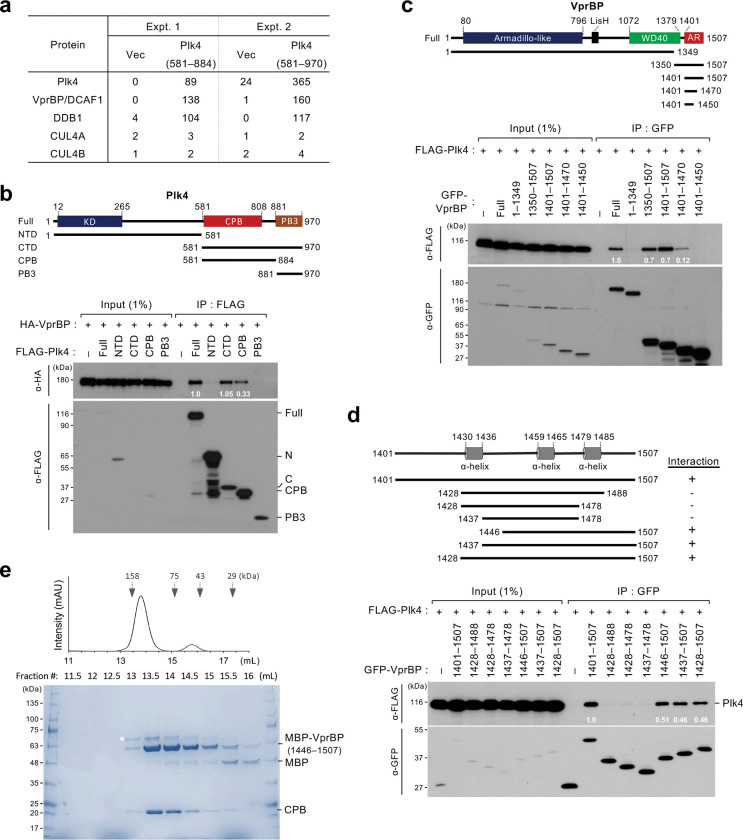
Direct interaction between VprBP AR and Plk4 CPB. **a,** Two independent mass spectrometry analyses showing that VprBP and DDB1 co-purified with the indicated Plk4 ligands. The peptide counts of the listed proteins co-purified with the ligands are shown. A ZZ-tagged Plk4 CPB (581–884) and a FLAG-tagged Plk4 CTD (581–970) were used as ligands for Expt. 1 and 2, respectively (see Methods for details). **b–d,** Coimmunoprecipitation analyses performed with HEK293T cells transfected with the indicated constructs. Residue numbers are shown in the schematic diagram. KD, kinase domain; CPB, cryptic polo-box domain; PB3, polo-box 3: NTD, N-terminal domain; CTD, C-terminal domain; WD40, WD40 domain; AR, Acidic region. Numbers in the immunoblots represent relative signal intensities. **e,** SEC profile and SDS-PAGE showing coeluting MBP-VprBP(1446–1507) and CPB(581–808) in the Coomassie Brilliant Blue-stained gel. Asterisk, contaminating protein.

To identify novel Plk4-binding proteins whose function could be influenced by HIV-1, we performed two independent affinity purification–mass spectrometry analyses using HEK293T cells expressing Plk4 CPB (581–884) or the entire C-terminal domain (CTD) (581–970). The results showed that both CPB and CTD effectively precipitated VprBP, a major HIV-1 Vpr-binding protein ^18^, and its associating DDB1, a subunit of VprBP-mediated E3 ligase complexes ^25,27,28,49,50^ (Fig. 2a). As expected, Plk4, which forms a homodimer ^37,51^, and other proteins known to interact/associate with Plk4 (such as Cep152, Cep192, PCNT, Cep63, Cep135, and Cep57) ^34–36,52–55^ were also copurified (Supplementary Table 1). Under these conditions, CUL4A and CUL4B, the subunits of E3 ubiquitin ligase complexes ^13,19,22,23^, were not significantly detected (Fig. 2a). Consistently, coimmunoprecipitation analyses carried out with transfected HEK293T cells showed that the full-length Plk4 efficiently coprecipitated VprBP under thymidine-treated (S-phase) or nocodazole-treated (G2/M-phase) conditions (Supplementary Fig. 2b). It also interacted with DDB1, albeit at a reduced level (Supplementary Fig. 2c).

In accord with the data in Fig. 2a, both CTD and CPB of Plk4, but not the N-terminal domain (NTD), effectively interacted with the full-length VprBP (Fig. 2b). In a reverse experiment, the AR (residues 1401–1507) of VprBP coprecipitated the full-length Plk4, whereas its partially deleted AR fragments (i.e., 1401–1470 and 1401–1450) exhibited a significantly compromised or an undetectable level, respectively, of Plk4 binding (Fig. 2c). VprBP(1446–1507) appeared to be sufficient for the VprBP–Plk4 interaction (Fig. 2d). Consistent with these observations, a recombinant His-MBP-fused VprBP(1446–1507) expressed in *E. coli* efficiently interacted with CPB (Supplementary Fig. 2d), and the two proteins coeluted from size-exclusion chromatography (SEC) (Fig. 2e). Taken together, these data suggest that VprBP forms a binary complex with Plk4 through the interaction between its AR and Plk4 CPB.

### HIV-1 Vpr, but not HIV-2 Vpx, forms a cooperative complex with VprBP and Plk4.

**Fig. 3.**
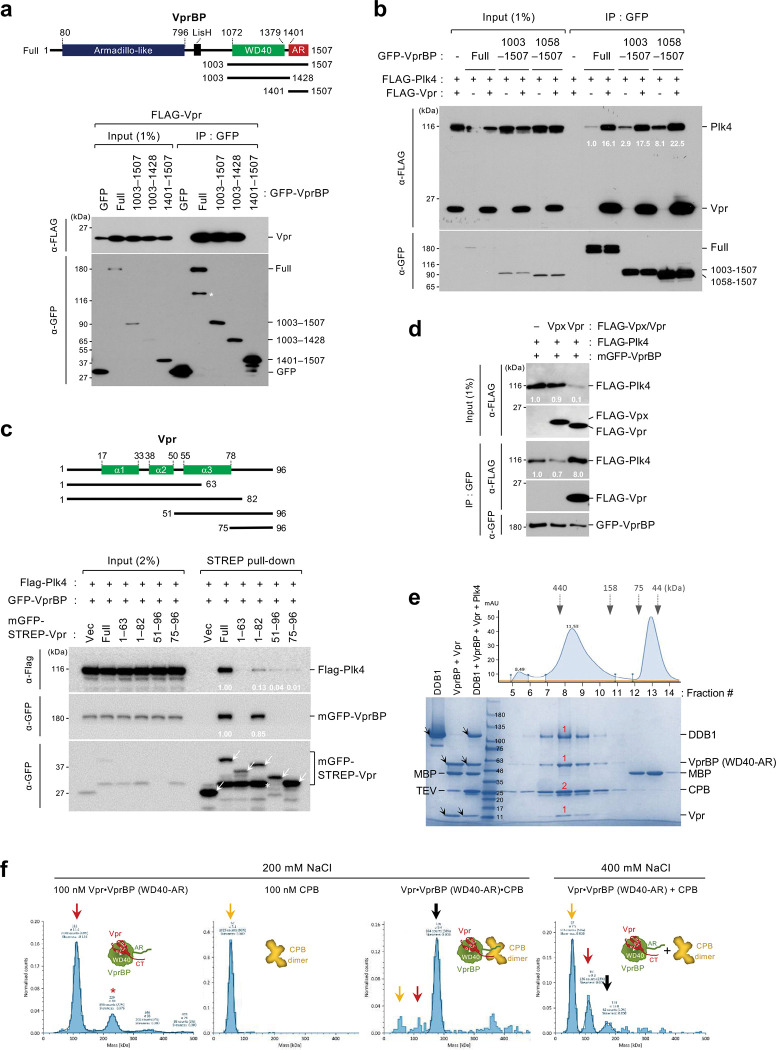
Vpr, but not Vpx, forms a ternary complex with VprBP WD40-AR and Plk4 CPB. **a–d,** Coimmunoprecipitation analyses performed with HEK293T cells transfected with the indicated constructs. Asterisk in (**a,c**), degradation product; arrows in (**c**), mGFP-STREP-containing ligands; numbers in (**b–d**), relative signal intensities. **e,** SEC profile and SDS-PAGE analysis demonstrating the purification of the DDB1•Vpr•VprBP WD40-AR(1057–1507)•Plk4 CPB(581–808) complex. Arrows, respective proteins loaded in each lane; red numbers, an estimated binding stoichiometry estimated from the Coomassie Brilliant Blue–stained protein intensity. **f,** Interferometric scattering mass spectrometry (iSCAMS) data showing the Vpr•VprBP WD4-AR•Plk4 CPB complex forming within 1 minute after mixing all components at 200 mM NaCl. The complex is sensitive to 400 mM NaCl (the 4^th^ panel). The 11-kDa Vpr, which binds tightly to VprBP, cannot be detected due to its small particle size. Red arrow, the Vpr•VprBP WD40-AR complex (red asterisk, a presumed dimer); yellow arrow, Plk4 CPB; thick black arrow, the Vpr•VprBP WD40-AR•Plk4 CPB complex.

As one of the main cellular binding targets for HIV-1 Vpr, VprBP is shown to bind to Vpr through the interactions between its WD40 domain and Vpr’s N-terminal and α3 region ^27^. As expected, Vpr interacted with the WD40-containing fragments but failed to interact with the AR(1401–1507) fragment (Fig. 3a). To determine whether Vpr influences the VprBP-Plk4 interaction, we performed coimmunoprecipitation analyses using various VprBP constructs containing the Vpr-binding WD40 domain. Remarkably, the coexpression of Vpr enhanced the level of VprBP-associated Plk4 severalfold (Fig. 3b and Supplementary Fig. 3a), suggesting that Vpr cooperatively generates a ternary complex with VprBP and Plk4. The catalytic activity of Plk4 was not required for Vpr-enhanced VprBP–Plk4 interaction (Supplementary Fig. 3b). In an experiment carried out with STREP-Vpr as a ligand, the ability of VprBP to augment the Vpr–Plk4 interaction was manifest (Supplementary Fig. 3c). In addition, the Vpr(1–82) truncate lacking the CT region exhibited a greatly impaired ability to interact with Plk4, although it largely maintained the level of VprBP binding (Fig. 3c). The C-terminal Vpr(51–96) and Vpr(75–96) fragments exhibited a capacity to interact with Plk4 at a low level (Fig. 3c). Unlike Vpr, several variants of Vpx exhibited either a low or undetectable level of binding to VprBP (Supplementary Fig. 3d) and failed to enhance the VprBP–Plk4 interaction (Fig. 3d). Further analysis with a recombinant Vpr showed that it directly interacted with CPB or CTD *in vitro* (Supplementary Fig. 3e). These findings and the data in Fig. 2 suggest that Vpr, VprBP (WD40-AR), and Plk4 CPB directly interact with one another and generate a cooperative ternary complex.

To investigate the nature of the ternary complex, SEC was carried out with purified proteins (i.e., Vpr, VprBP[1057–1507; WD40-AR], Plk4 CPB, and DDB1). Analysis of fractionated samples suggests the formation of a ternary Vpr•VprBP•Plk4 complex with an approximately 1:1:2 stoichiometry (Fig. 3e). (Plk4 functions as a homodimer ^37,51^.) DDB1, which is shown to bind to VprBP ^27^, also copurified with the ternary complex (Fig. 3e). Further analyses with interferometric scattering mass spectrometry (iSCAMS), which detects protein–protein interactions in a real-time, revealed that, at 200 mM NaCl, the VprBP (WD40-AR)•Vpr complex (Fig. 3f, 1^st^ panel, red arrow) generated a ternary complex with Plk4 CPB (Fig. 3f, 3^rd^ panel, green arrow) immediately after mixing (in less than a minute). At 400 mM NaCl, however, the ternary complex became largely dissociated (Fig. 3f, 4^th^ panel), presumably because the AR region, which is heavily enriched in Asp and Glu residues with the calculated pI of 2.4, cannot stably interact with the basic CPB (pI of 9.1) in a high-ionic-strength environment.

### Plk4 is not the target of the Vpr•VprBP-mediated E3 ubiquitin ligase.

Various studies have shown that Vpr forms a complex with a cullin-4-RING E3 ubiquitin ligase (i.e., the CRL4-VprBP-Vpr complex) and regulates multiple intracellular target proteins through proteasomal degradation ^13,19,22,23^. However, overexpression of Vpr, VprBP, or both did not detectably alter Plk4 stability, whereas it effectively induced degradation of a previously characterized substrate, Uracil DNA Glycosylase-2 (UNG2) ^49^ (Supplementary Fig. 3f,g). In addition, while depletion of β-TrCP, a known F-box protein for Plk4 ^56^, increased the steady-state level of Plk4, depletion of VprBP failed to noticeably change the level of Plk4 (Supplementary Fig. 3h). In *in vitro* ubiquitination assays carried out with purified proteins, Vpr did not appear to influence the level of ubiquitinated Plk4 (Supplementary Fig. 3i). Under the same conditions, however, ubiquitination of UNG2 was enhanced even by the Vpr(1–79) form lacking the CT (Supplementary Fig. 3i, bottom), as reported previously ^49^. These observations suggest that the binding of Vpr and VprBP may not prompt proteasomal degradation of Plk4.

### Vpr and VprBP colocalize with Plk4 and promote Plk4’s ring-to-dot conversion around a centriole.

**Fig. 4.**
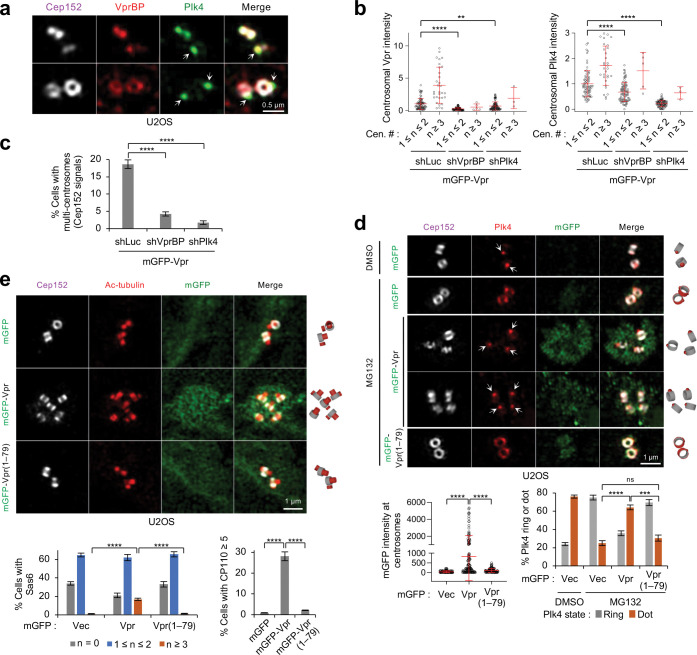
Vpr enforces a dot-state Plk4 and centriole overduplication in its CT-dependent manner. **a,** 3D-SIM image showing the localization pattern of VprBP around a centriole in U2OS cells. Arrows, dot-state Plk4 signals. **b,c,** Quantification of centrosome-localizing Vpr and Plk4 signals (**b**) and Vpr-induced, over-duplicated centrosomes (**c**) after treating the mGFP-Vpr-expressing U2OS cells with the indicated shRNA. Images used for quantifications in (**b,c**) are provided in Supplementary Fig. 4c. Data were obtained from three independent experiments. For (**b**), per experiment: n ≥ 35 for shLuc (total n = 109); n ≥ 30 for shVprBP (total n = 91); n = 30 for shPlk4 (total n = 90). Bars, mean of n ± s.d. For (**c**), per experiment: n ≥ 480 for shLuc (total n = 1528); n ≥ 441 for shVprBP (total n = 1342); n ≥ 447 for shPlk4 (total n = 1389). Bars, mean of three experiments ± s.d. **P < 0.01; ****P < 0.0001 (unpaired two-tailed *t*-test). **d,** 3D-SIM images obtained from the cells in Supplementary Fig. 4e show over-duplicated centrosomes by Vpr WT but not Vpr(1–79) lacking the CT. To clearly distinguish Plk4’s ring-state vs. dot-state localization in (**d**), cells were treated with MG132 for 3 hours. Arrows, dot-state Plk4. Schematic diagrams (right) show the localization patterns of Cep152 and Plk4 signals. Quantified data (graphs) were obtained from three independent experiments. For mGFP intensities measured per experiment: n ≥ 37 for mGFP (total n = 113); n ≥ 41 for mGFP-Vpr (total n = 126); n ≥ 30 for mGFP-Vpr(1–79) (total n = 96). Bars, mean of n ± s.d. For Plk4 ring or dot quantified per experiment: n ≥ 219 for mGFP/DMSO (total n = 665); n ≥ 150 for mGFP/MG132 (total n = 494); n ≥ 180 for mGFP-Vpr/MG132 (total n = 692); n ≥ 131 for mGFP-Vpr(1–79)/MG132 (total n = 411). Bars, mean of three experiments ± s.d. ***P < 0.001; ****P < 0.0001 (unpaired two-tailed *t*-test); ns, not significant. **e,** Representative 3D-SIM images for cells immunostained with an anti-acetylated-tubulin antibody (top) and quantification of Sas6 and CP110 signals (graphs) using images shown in Supplementary Fig. 4f. Schematic diagrams (right) are shown for Cep152 and acetylated tubulin signals. Quantification was performed from three independent experiments. For Sas6 counts per experiment: n ≥ 407 for mGFP (total n = 1421); n ≥ 411 for mGFP-Vpr (total n = 1332); n ≥ 418 for mGFP-Vpr(1–79) (total n = 1351). For CP110 counts per experiment: n ≥ 417 for mGFP (total n = 1369); n ≥ 453 for mGFP-Vpr (total n = 1418); n ≥ 471 for mGFP-Vpr(1–79) (total n = 1534). Bars, mean of three experiments ± s.d. ****P < 0.0001 (unpaired two-tailed *t*-test).

Consistent with the previous findings ^28,57^, VprBP localized to the nucleus and centrosomes (Supplementary Fig. 4a) with its signals found at or near a region where Cep152 localizes (Fig. 4a). Since VprBP AR interacted with Plk4 CPB (Fig. 2), we investigated whether VprBP influences Plk4’s pericentriolar localization in an AR-dependent manner. In U2OS cells, depletion of endogenous VprBP by RNAi mildly reduced the number of the dot-state Plk4, and the expression of RNAi-insensitive VprBP, but not the VprBPΔAR mutant (residues 1–1427), rescued this defect (Supplementary Fig. 4b). This suggests that, albeit at a low degree, the AR-dependent VprBP function contributes to the capacity of Plk4 to dynamically relocalize to the procentriole assembly site (the dot state in Supplementary Fig. 4b) from around a centriole (the ring state in Supplementary Fig. 4b).

In a related experiment, depletion of either VprBP or Plk4 drastically reduced centrosome-localized Vpr signals (Fig. 4b, left, and Supplementary Fig. 4c). This aligns with the observation that Vpr interacts with VprBP and Plk4 (Fig. 3 and Supplementary Fig. 3a–e). VprBP depletion also significantly lowered the level of Plk4 recruited to centrosomes (Fig. 4b, right). This observation would not be expected if VprBP were to promote Plk4 degradation. Not surprisingly, both VprBP and Plk4 were required for overexpressed Vpr to induce multiple centrosomes (judged by the Cep152 signals, whose localization tightly correlated with γ-tubulin signals throughout the cell cycle) (Fig. 4c and Supplementary Fig. 4c). The Vpr K27M mutant relieved of Vpr-imposed G2 arrest ^58^ exhibited an undiminished capacity to bind to VprBP and Plk4 and induce multiple signals of a centriolar scaffold, Sas6 ^59^ (Supplementary Fig. 4d). This suggests that Vpr can induce multiple centrosomes independently of Vpr-induced G2 arrest, as reported previously ^16^.

Next, to determine whether Vpr alters Plk4’s localization dynamics and Plk4-dependent centriole biogenesis, we performed comparative immunostaining analyses using cells expressing a monomeric GFP (mGFP)-Vpr or mGFP-Vpr(1–79) lacking the Plk4-binding C-terminal 17 residues (Supplementary Fig. 4e). Notably, mGFP-Vpr alone effectively localized to the pericentriolar material (PCM) region, often showing a “nebulous” appearance that encompasses multiple dot-like Plk4 signals (Fig. 4d and Supplementary Fig. 4f). In contrast, the Vpr(1–79) mutant localized poorly to centrosomes and failed to significantly induce the dot-like Plk4 signals (Fig. 4d and Supplementary Fig. 4f). Consistent with this finding, a substantial fraction of cells expressing Vpr, but not the Vpr(1–79) mutant, exhibited multiple acetylated tubulins (Fig. 4e) and significantly increased the number of Sas6 ^59,60^ and CP110 signals ^29,30^ (Fig. 4e and Supplementary Fig. 4f). mGFP-Vpr induced multiple centrosomes independently of the cell cycle (Supplementary Fig. 4g). These data suggest that Vpr CT-dependent interaction with Plk4 heightens Plk4’s ring-to-dot relocalization and centriole overduplication, ultimately generating multiple centrosomes.

### Both Vpr CT and VprBP AR are critical for Vpr•VprBP•Plk4-mediated centriole overduplication.

**Fig. 5.**
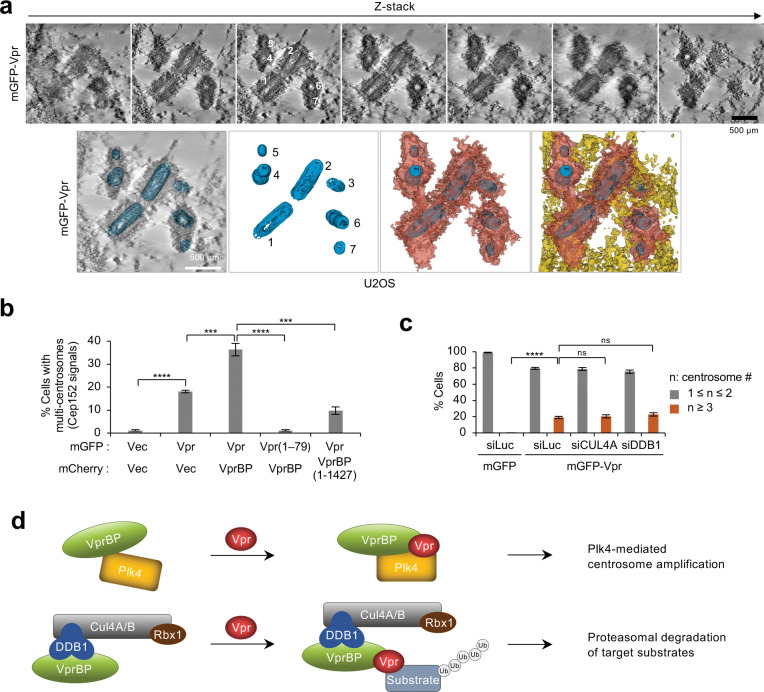
Vpr CT and VprBP AR are required for Vpr•VprBP•Plk4-mediated centriole overduplication independently of the CUL4-DDB1 ubiquitin ligase. **a,** TEM tomography and segmentation of centrioles for a U2OS cell expressing mGFP-Vpr. Serial TEM images are provided at different z-sections showing mGFP-Vpr-induced multiple centrioles (numbered from 1 to 7). Centrioles (blue) and their directly associated (red) and surrounding (yellow) cellular materials are shown. **b**, Quantification of immunostained cells in Supplementary Fig. 5a, stably expressing the indicated Vpr and VprBP constructs using a lentiviral system. Results were obtained from three independent experiments [per experiment, n ≥ 149 for mGFP + mCherry (total n = 459); n ≥ 166 for mGFP-Vpr + mCherry (total n = 530); n ≥ 143 for mGFP-Vpr + mCherry-VprBP (total n = 453); n ≥ 143 for mGFP-Vpr(1–79) + mCherry-VprBP (total n = 446); n ≥ 134 for mGFP-Vpr + mCherry-VprBP(1–1427) (total n = 439)]. Bars, mean of three experiments ± s.d. ***P < 0.001; ****P < 0.0001 (unpaired two-tailed *t*-test). **c,** Quantification of immunostained cells in Supplementary Fig. 5b after depletion of CUL4A, the cytoplasm-localized form ^61^, or DDB1 by RNAi. Quantification was performed from three independent experiments [per experiment, n ≥ 609 for mGFP/siLuc (total n = 2132); n ≥ 672 for mGFP-Vpr/siLuc (total n = 2174); n ≥ 624 for mGFP-Vpr/siCUL4A (total n = 1933); n ≥ 623 for mGFP-Vpr/siDDB1 (total n = 1927)]. Bars, mean of three experiments ± s.d. ****P < 0.0001 (unpaired two-tailed *t*-test); ns, not significant. **d,** Schematic diagrams illustrating how Vpr hijacks two distinct cellular complexes—one that overdrives the Plk4-dependent centriole duplication event (top) and the other that promotes the VprBP-mediated E3 ligase activity (bottom).

To corroborate the formation of multiple centrioles in mGFP-Vpr-expressing cells, we performed transmission electron microscope (TEM) tomography as described in the Method. The result showed that some cells exhibit up to 7 centrioles, often in a clustered region (Fig. 5a). This observation is in line with the data shown in Fig. 4b–e.

Since Vpr and VprBP are required for inducing multiple centrioles (Fig. 4c), we then investigated the significance of forming a ternary Vpr•VprBP•Plk4 complex in Fig. 3 in deregulating centriole duplication by immunostaining U2OS cells stably expressing the indicated Vpr and/or VprBP constructs. Under the conditions where exogenous Vpr and VprBP were expressed at comparable levels, Vpr robustly induced centrosome overduplication and VprBP further augmented it (Fig. 5b and Supplementary Fig. 5a). Both Vpr(1–79; ΔCT) and VprBP(1–1427; ΔAR) mutants defective in Plk4 binding failed to promote this event (Fig. 5b and Supplementary Fig. 5a). Thus, Vpr cooperates with VprBP to induce centrosome overduplication in a manner that requires the Plk4-binding Vpr CT and VprBP AR regions.

Since DDB1 interacting with VprBP coprecipitated with CPB and Plk4 (Fig. 2a and Supplementary Fig. 2c), we examined whether DDB1 or its upstream CUL4A, a cytoplasm-localized cullin family member of ubiquitin ligases ^61^, is required for Vpr-induced centrosome overduplication. In immunostaining analyses performed with cells expressing mGFP-Vpr, depletion of either CUL4A or DDB1 did not significantly influence the degree of mGFP-Vpr-induced centrosome overduplication (Fig. 5c and Supplementary Fig. 5b). These data further suggest that the Vpr•VprBP•Plk4 complex induces centrosome overduplication independently of the VprBP-mediated E3 ligase activity (Fig. 5d and Supplementary Fig. 3f–i).

### HIV-1 Vpr, but not the Plk4 binding–defective Vpr(1–79) mutant, induces centrosome overduplication in various CD4^+^ cells.

**Fig. 6.**
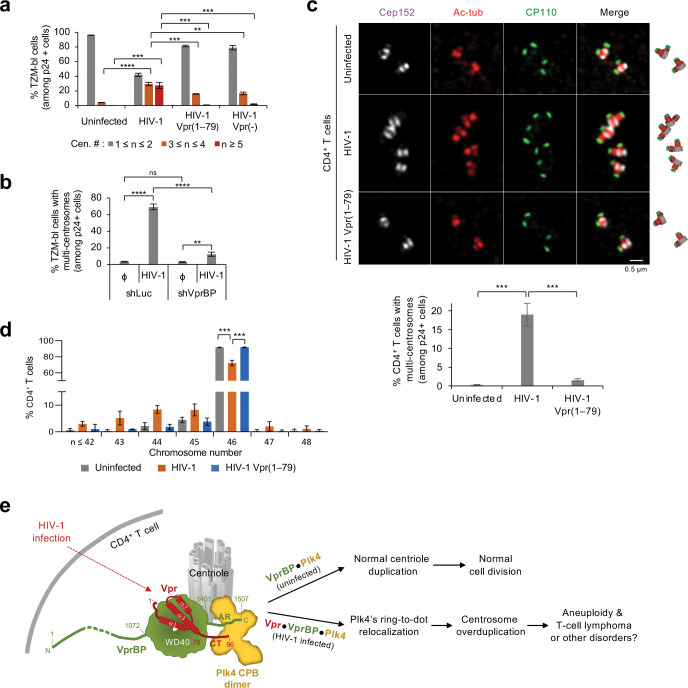
Induction of centrosome amplification and aneuploidy by HIV-1 WT but not the Plk4 binding^–^defective HIV-1 Vpr(1–79) mutant. **a**, Quantification of immunostained CD4^+^ TZM-bl cells infected with the indicated HIV-1 WT or Vpr mutants (representative images shown in Supplementary Fig. 6a). Centrosomes (marked by Cep152 signals) were counted among the HIV-1-infected p24^+^ cells. HIV-1 Vpr(−) denotes a Vpr-null mutant. Results were quantified from three independent experiments [per experiment, n ≥ 213 for uninfected cells (total n = 707); n ≥ 202 for HIV-1 (total n = 621); n ≥ 246 for HIV-1 Vpr(1–79) (total n = 795); n ≥ 204 for HIV-1 Vpr(2212) (total n = 637)]. Bars, mean of three experiments ± s.d. **P < 0.01; ***P < 0.001; ****P < 0.0001 (unpaired two-tailed *t*-test). **b,** Quantification of cells with multi-centrosomes (i.e., Cep152 signals) performed after infecting them with HIV-1 and silencing them for control luciferase (shLuc) or VprBP (shVprBP). Counts were among the p24^+^ population (representative images provided in Supplementary Fig. 6c) from three independent experiments. Per experiment, n ≥ 232 for uninfected/shLuc (total n = 737); n ≥ 209 for HIV-1/shLuc (total n = 656); n ≥ 222 for uninfected/shVprBP (total n = 713); n ≥ 245 for HIV-1/shVprBP (total n = 750)]. Bars, mean of three experiments ± s.d. **P < 0.01; ****P < 0.0001 (unpaired two-tailed *t*-test); ns, not significant. **c,** 3D-SIM and quantification of immunostained primary CD4^+^ T cells purified from healthy PBMCs, infected with HIV-1 WT or Vpr(1–79) mutant for 8 h, and cultured on the anti-CD3 and anti-CD28 antibody–coated plate for 4 days. Schematic diagrams (right) are shown for merged images. Quantified centrosome numbers (marked by Cep152 signals) (graph) were obtained from three independent experiments. Per experiment, n ≥ 790 for uninfected cells (total n = 2925); n ≥ 535 for HIV-1 (total n = 1996); n ≥ 520 for HIV-1 Vpr(1–79) (total n = 2323)]. Bars, mean of three experiments ± s.d. ***P < 0.001 (unpaired two-tailed *t*-test). **d,** Quantification of chromosome numbers for primary CD4^+^ T cells infected with the indicated viruses for 8 hours and cultured on the anti-CD3 and anti-CD28 antibody–coated plate for 4 days. Results were obtained from three independent experiments carried out with CD4^+^ T cells purified from three healthy individuals. Representative chromosome spread images used for quantification are provided in Supplementary Fig. 6e. Per experiment, n ≥ 87 for uninfected cells (total n = 288); n ≥ 88 for HIV-1 (total n = 299); n ≥ 97 for HIV-1 Vpr(1–79) (total n = 328). Bars, mean of three experiments ± s.d. ***P < 0.001 (unpaired two-tailed *t*-test). **e,** Model illustrating how HIV-1 Vpr generates the Vpr•VprBP•Plk4 complex and induces centrosome amplification and aneuploidy, a cause of cancer development ^45,46,67^. Under unperturbed conditions, VprBP binds to Plk4 through its C-terminal AR and contributes to Plk4-dependent centriole duplication and normal cell division. When HIV-1 infects CD4^+^ T cells, its accessory protein, Vpr, forms a ternary complex with VprBP and Plk4 and induces Plk4-mediated centrosome overduplication and aneuploidy (rather than degrading Plk4 through the VprBP-mediated E3 ligase activity). Given that integration of HIV-1 proviruses into oncogenes, such as STAT3 and LCK, can promote the development of T-cell lymphomas ^5^, HIV-1 may induce oncogenesis by driving both provirus integration–mediated oncogene activation and Vpr-mediated deregulation of the centriole duplication machinery.

We examined the effect of Vpr expression on centriole duplication in multiple CD4^+^ cells infected with HIV-1 pseudoviruses (see Methods). In TZM-bl cells (derived from HeLa cells) or CEM-SS cells (derived from CEM CD4^+^ T cells), neither HIV-1 wild type (WT) nor its respective Vpr mutants noticeably altered the levels of various components critical for centriole biogenesis (Supplementary Fig. 6a,b). Under these conditions, while HIV-1 WT drastically induced cells with multiple centrosomes (i.e., greater than two Cep152 signals counted among the p24^+^ population), its respective Vpr(1–79) and Vpr(−) mutants failed to do so (Fig. 6a and Supplementary Fig. 6a,b). These data, which corroborate the results obtained from U2OS cells (Figs. 4d,e, 5a), suggest that eliminating Vpr CT-dependent Plk4 interaction is sufficient to abrogate Vpr-induced centrosome amplification in CD4^+^ cells. In a related experiment, HIV-1 Vpr-induced, overduplicated centrosomes (marked by pericentriolar Cep152) were nearly annihilated by the depletion of VprBP (Fig. 6b and Supplementary Fig. 6c). This reinforces that VprBP is required for Vpr-induced centrosome amplification, as shown in Fig. 4c. VprBP may serve as the critical scaffold for the ternary complex, which enables Vpr CT to trigger Plk4-mediated centrosome duplication.

Next, we investigated whether HIV-1 Vpr CT-dependent centrosome amplification is sufficient to induce aneuploidy, a condition that could drive oncogenesis ^10,44–46^. To this end, purified CD4^+^ T cells from healthy individuals were stimulated on an anti-CD3 and anti-CD28 antibody–coated plate for one day, infected with HIV-1 WT or its respective HIV-1 Vpr(1–79) mutant for 8 hours, and additionally cultured for 4 days before analyses. In line with the data described above, the primary T cells infected with HIV-1 WT, but not its respective Vpr(1–79) mutant, exhibited centrosome amplification (marked by Cep152, acetylated tubulin, and CP110 signals) in approximately 19% of the p24^+^ population (Fig. 6c and Supplementary Fig. 6d). Consistently, chromosome spread analyses revealed that infection with HIV-1 WT, but not the Vpr(1–79) mutant, increased the fraction of aneuploid cells by approximately 20% (mean of three experiments carried out with the primary CD4^+^ T cells purified from as many healthy subjects) (Fig. 6d and Supplementary Fig. 6e). These findings, along with the data shown in Supplementary Fig. 3e, suggest that the Vpr CT-dependent interaction with Plk4 CPB underlies the induction of aneuploidy in these cells.

## Discussion

People living with HIV-1 are at a greatly increased risk of developing non-Hodgkin’s lymphoma ^62,63^. Although not as prevalent as B-cell lymphomas, a study analyzing the AIDS–Cancer Match Registry of more than 300,000 adults with AIDS shows an approximately 15-fold increase in T-cell lymphomas (24-fold increase in the case of peripheral T-cell lymphoma) ^64^. Notably, the risk of developing non-Hodgkin’s lymphoma is far greater in individuals with HIV-1 than in immunosuppressed transplant recipients ^63^. This suggests that factor(s) other than HIV-1-induced immune deficiency promotes HIV-1-associated cancers.

How T-cell lymphomas can arise under conditions where HIV-1 can progressively destroy them remains unclear. A body of evidence suggests that HIV-1 proviruses integrated into several oncogenes, such as signal transducer and activator of transcription 3 (*STAT3*) and lymphocyte-specific protein tyrosine kinase (*LCK*), thus paving the way for developing T-cell lymphomas ^5,65,66^. Fascinated by the finding that centrosomes are amplified in the primary CD4^+^ T cells from people living with HIV-1, here we explored whether an HIV-1-encoded protein(s) can directly deregulate host cellular processes that can promote oncogenesis. In this regard, given that aneuploidy is considered a cause of cancer development ^45,46,67^, our discovery of a ternary Vpr•VprBP•Plk4 complex and the ability of the complex to potentiate Plk4-mediated centriole duplication and induce aneuploidy are significant (Fig. 6e). Vpr expressed from an integrated HIV-1 provirus could contribute to the development of T-cell lymphoma, either alone or in combination with the event triggered by the proviral DNA integration itself. In addition, since a substantial level of Vpr (10 pg/mL–10 ng/mL of blood) is present in the blood of people living with HIV-1 ^68–70^ and because Vpr exhibits membrane-penetrating properties ^71^, Vpr circulating in the bloodstream of a system could give rise to centrosome abnormality–associated tumorigenesis in cells other than CD4^+^ T cells.

Our data showed that Vpr’s capacity to interact with both VprBP WD40 and Plk4 CPB is essential to bolster the ability of the VprBP•Plk4 complex to induce centriole biogenesis. Not surprisingly, when coexpressed, all three proteins proficiently generated colocalized signals in a manner that requires the Vpr CT(80–96) region. Consistent with this finding, HIV-1 WT, but not the Plk4 binding–defective VprΔ(80–96) mutant, potently induced centrosome overduplication and aneuploidy in primary CD4^+^ T cells purified from PBMCs of healthy human subjects (Fig. 6c,d). These findings suggest that targeting the Vpr C-terminal motif could be an attractive strategy to antagonize the function of the Vpr•VprBP•Plk4 complex and thereby reduce the occurrence of HIV-1-associated cancers.

Centrosome amplification, a cause of chromosomal instability and aneuploidy ^10^, is prevalent among hematological malignancies ^6–9^, and its degree of abnormalities closely correlates with tumor grades ^72,73^. As a master regulator of centriole duplication, deregulated Plk4 activity appears to be tightly associated with oncogenesis ^10,44,74^. A modest elevation of Plk4 level (< 2-fold) is sufficient to induce centrosome amplification and aneuploidy that lead to the generation of various spontaneous tumors in a mouse model ^48^. Here we demonstrated that, through the formation of the Vpr•VprBP•Plk4 complex, Vpr deregulates Plk4’s functionality and induces centrosome amplification and aneuploidy in HIV-1-susceptible CD4^+^ T cells. In light of a recent view that HIV-1 provirus integration into oncogenes could lead to developing T-cell lymphomas ^5^, this study may offer an unexplored avenue for investigating the molecular mechanism underlying how comorbid cancers arise in people living with HIV-1.

## Methods

### Plasmid constructs.

The control vector pHR.J-ZZ-TEV and pHR.J-ZZ-TEV-Plk4(581–884) constructs (pKM4283 and pKM4285) were generated by inserting a HindIII (end-filled)-BamHI or HindIII (end-filled)-XhoI fragment into the pHR′.J-CMV-SV-puro vector ^75^ digested by SamI and BamHI or SmaI or SalI, respectively. The FLAG-fused Plk4(581–970) construct (pKM4591) is described by Park et al. (*Nat Comm,* 2019,). The various FLAG-fused Plk4 constructs (pKM3445, pKM3448, and pKM3506–pKM3509) and the HA-tagged Plk4 construct (pKM3855) were reported previously ^36,37^. The construct was generated similarly by using the pCI-neo-HA vector (pKM1209). The HA-fused VprBP construct (pKM4358) was constructed by inserting a PmeI-XhoI fragment into the pCI-neo-HA vector (pKM1209) at the corresponding sites. Various GFP-fused VprBP clones (pKM4543–pKM4548, pKM5705, pKM5684, pKM5686, pKM5721–pKM5723, pKM5805, pKM4617, pKM4836) were constructed by inserting PmeI-NotI fragments into the pCI-neo-GFP vector (pKM3828) digested by the same enzymes. The constructs expressing VprBP (pKM4630) and its truncates (pKM5284, pKM5412, and pKM5283) were generated by inserting a PmeI-XhoI or PmeI-NotI fragment, respectively, into the pKM2795 vector digested with corresponding enzymes. To generate the lentivirus-based, the mCherry-tagged VprBP full length or VprBP(1–1427) (pKM7574 or pKM7794), the EcoRI-SalI fragments were inserted into the pHR′.J-CMV-SV-puro (pKM2994) digested by the same enzyme.

The FLAG-tagged Vpr (pKM5330) and HA-tagged Vpr WT or its K27M mutant (pKM5523 or pKM5755) were generated by inserting a PmeI-XhoI fragment into the pKM2795 and pKM1209 vector, respectively. Unless otherwise indicated as gift constructs (see Supplementary Table 1), all the Vpr constructs generated in this study are derived from the infectious molecular clone pNL4–3 ^76^. The open reading frame (ORF) of Vpr in pKM5330 contains Y15, S28, N41, and R85 residues. It is identical in the primary sequence to the Vpr constructs provided as gifts by Jae-Il Park (MD Anderson Cancer Center, TX), Angela M. Gronenborn (University of Pittsburgh, PA; pKM4759), and Jeremy Luban (University of Massachusetts Medical School, MA). The FLAG-Vpr construct (pKM4758; Lai strain containing H15, N28, G41, and Q85 residues) was provided by Michael Emerman (Fred Hutchinson Cancer Center, WA). Both Vpr variants showed similar cooperativity in forming a ternary complex with VprBP and Plk4 (Supplementary Fig. 3c). Various Vpx constructs (pKM4753–pKM4757) were provided by Monsef Benkirane (Institut de Génétique Humaine, Montpellier, France) and Michael Emerman (Fred Hutchinson Cancer Center, WA). Various mGFP-STREP-TEV-fused Vpr clones (pKM7653–pKM7655 and pKM7666–pKM7667) were constructed by inserting AscI-BamHI fragments into the pHR′.J-CMV-SV-puro-mGFP-STREP-TEV vector (pKM7652) digested by the same enzymes. Lentiviral mGFP-Vpr constructs (pKM7618, pKM7792) were generated by inserting an AscI-BamHI fragment containing Vpr WT or Vpr(1–79) into the pHR′.J-CMV-SV-puro-mGFP (pKM7410) vector digested by the corresponding enzymes.

The HA-fused DDB1 construct (pKM4354)was generated by cloning a PmeI-NotI fragment into the pKM1209 vector digested by the same enzymes. The HA-tagged Cep57, HA-tagged Cep63, and FLAG-tagged Cep152 constructs (pKM1234, pKM1235, and pKM2809) were described previously ^55,77^.

pLKO.1 hygro-shLuc, hygro-shPlk4, and hygro-shVprBP (pKM7743, pKM7744, and pKM7746) were generated by inserting an AgeI-EcoRI fragment into the pLKO.1 hygro vector (Addgene, Watertown, MA).

An *env*-deleted variant of pNL4–3, pNLenv1 (pKM7595), was generated as follows: An EcoRI/BamHI fragment from pNL43 was subcloned into pUC18. The plasmid was then digested with KpnI and BglII, the overhanging ends filled-in with DNA polymerase and the plasmid religated. This operation created a 1264 bp out-of-frame deletion in the *env* gene. The shortened EcoRI/BamHI fragment was then cloned back into pNL4–3, resulting in pNLenv1. To create a Vpr(−) variant of pNLenv1 (pKM7628), a SphI/BamHI fragment containing parts of *gag*, all of *pol* and *Vif*, and parts of *vpr* genes was subcloned into pUC18. A stop codon was then introduced into the vpr open reading frame after residue 2 using PCR-based mutagenesis. This operation did not affect the overlapping *vif* gene. The mutated fragment was then cloned back into the pNLenv1 backbone via the unique PflMI, BamHI restriction sites. The env-deleted pNL4–3env1 Vpr(1–79) (pKM8247) was generated by engineering an EcoRI-SalI fragment containing a stop codon after S79 and replacing the EcoRI-SalI fragment in pKM7595. The construct was confirmed by sequencing.

The His-MBP-TEV-CPB(581–808) construct (pKM3677) was reported previously ^37^. The His-MBP-M-CTD(468–970) (pKM7608) was generated by replacing the Plk4 CPB fragment in pKM3677 with the Plk4 M-CTD(468–970) fragment digested with NdeI and XhoI. The His-MBP-TEV-Plk4 WT and its respective K41M mutant (pKM4601 and pKM4602, respectively) were generated similarly to pKM7608 by inserting the corresponding fragments at the NdeI and XhoI sites. The pETDuet-1-His-MBP-TEV-based construct, dually expressing VprBP(1446–1507) and CPB(581–808) (M6934), was generated by inserting respective PmeI-NotI and NdeI-XhoI fragments into the corresponding enzyme sites. The NusA-Vpr-His (pKM6931) and UNG2 were described previously ^27,49^.

The pTriEx-4 (Addgene)-based insect construct dually expressing His-MBP-TEV-VprBP(1021–1507) and Vpr (pKM7669) was generated by inserting the entire DNA sequence synthesized by GenScript Biotech (Piscataway, NJ) at the AscI and XhoI sites. An additional SV40 poly(A) sequence, a p10 promoter, and a ribosome-binding site sequence were inserted before the Vpr ORF to ensure independent Vpr expression. The insect construct expressing DDB1 was reported previously ^49^.

All the constructs used for this study are summarized in Supplementary Table 1.

### Cell culture.

U2OS cells (for imaging analyses) and HEK293T cells (for lentivirus production and coimmunoprecipitation analysis) were cultured as recommended by the American Type Culture Collection (ATCC). Two CD4^+^ cell lines (TZM-bl [ARP-8129; contributed by John C. Kappes and Xiaoyun Wu] and CEM-SS [ARP-776; contributed by Peter L. Nara]) were obtained through the NIH HIV Reagent Program, Division of AIDS, National Institute of Allergy and Infectious Diseases, NIH. U2OS cells were cultured in McCoy’s 5A (Invitrogen), HEK293T and TZM-bl cells were cultured in Dulbecco’s Modified Eagle Medium (Thermo Fisher Scientific), and CEM-SS and T cells were cultured in RPMI 1640 Medium (Thermo Fisher Scientific). All media were supplemented with 10% fetal bovine serum (FBS). Where indicated, cells were treated with cycloheximide (100 μg/mL) to examine the half-life of cellular proteins after blocking their translational elongation capacity. In addition, cells were treated with 10 μM of MG132, a reversible proteasome inhibitor ^78^, for 3 or 6 hours, as indicated, to stabilize cellular proteins by inhibiting the degradation of ubiquitin-conjugated proteins.

To isolate human CD4^+^ T cells, apheresis from healthy individuals was collected from the NIH blood bank. Cells were layered on Ficoll-Paque premium (Fisher Scientific) and centrifuged at 1,600 rpm for 20 minutes at room temperature. The buffy coat containing PBMCs was collected and washed, and CD4^+^ T cells were purified using the EasySep human CD4^+^ T-cell isolation kit (STEMCELL Technologies). T cells were then stimulated by culturing them on anti-CD3 (2 μg/mL) and anti-CD28 (2 μg/mL) antibody–coated plates in complete RPMI 1640 Medium supplemented by 10% FBS.

Sf9 insect cells were cultured in Grace’s Insect Medium (Thermo Fisher Scientific) supplemented with 10% FBS, 1% Antibiotic-Antimycotic (Thermo Fisher Scientific), and 0.1% Pluronic F-68 (Sigma-Aldrich). Exponentially growing cells were incubated in a temperature-controlled orbital shaker at 150 rpm and 27 °C. For protein expression, Tni-FNL (FNL Hi5) cells were cultured in Sf-900 III SFM (Thermo Fisher Scientific) and shaken at 150 rpm at 27 °C. Cells were passaged when their counts were > 4 × 10^6^ viable cells/mL. After infection with viruses, the cells were cultured for additional 2.5 days and harvested for protein purification.

### Transfection.

For transfection, the calcium phosphate coprecipitation method was used for lentivirus production, while Lipofectamine RNAiMAX (Thermo Fisher Scientific) was used for short interfering RNA (siRNA)-based gene silencing. Transfection using PEI MAX (Polysciences) was implemented to express proteins for immunoprecipitation.

Endogenous VprBP, Plk4, DDB1, CUL4A, or bTrCP were depleted by either transfecting cells with an siRNA targeting a specific gene of interest or infecting them with lentiviruses expressing the respective short hairpin RNA (shRNA). All the siRNAs and shRNAs used for this study are listed in Supplementary Table 2.

BacMagic Transfection Kit (MilliporeSigma) was used for virus production in Sf9 cells according to the manufacturer’s protocol.

### Lentivirus and cell line generation.

Lentiviruses expressing the gene of interest were produced by cotransfecting HEK293T cells with pHR′-CMVΔR8.2Δvpr, pHR′-CMV-VSV-G (protein G of vesicular stomatitis virus), and the respective pHR′.J-CMV-SV-puro- or pLKO.1 hygro-based constructs listed in Supplementary Table 1. The resulting viruses were used as described previously ^79^. Where indicated, cells were additionally infected with lentiviruses expressing shRNAs (listed in Supplementary Table 2) to deplete the respective endogenous proteins before further analysis. To stably maintain the expression of lentivirus-encoded constructs and/or depletion of target proteins, cells were continuously cultured under puromycin (2 μg/mL) and/or hygromycin (300 μg/mL) during the entire experimental period.

### Pseudo-HIV-1 production and infection.

Pseudo-HIV-1 WT and Vpr mutants were produced as described previously ^80^. In short, HEK293T cells were cotransfected with pNL4–3env1, pNL4–3env1 Vpr(1–79), or pNL4–3env1 Vpr(−) with pCMV-VSVG at a 9:1 ratio. Forty-eight hours after transfection, viruses were collected and kept frozen until use.

TZM-bl and CEM-SS cells were infected with the viruses above for 12 hours and the resulting cells were cultured in a fresh medium for 2.5 days (for TZM-bl) or 1.5 days (for CEM-SS) before immunostaining and immunoblotting analyses.

Primary CD4^+^ T cells were infected 24 hours after stimulating the cells on an anti-CD3 (2 μg/mL) and anti-CD28 (2 μg/mL) antibody–coated plate. After removing supernatant viruses 8 hours postinfection, the resulting cells were plated again on the antibody-coated plate and cultured for four days before subjecting them to immunostaining and chromosome spread analyses. Immunostaining analysis with anti-p24 antibody showed that approximately 10% of T cells were typically infected under our experimental conditions.

### Immunostaining.

Immunostaining was performed as described previously ^36^. In brief, U2OS or TZM-bl cells were grown on poly-L-lysine (Sigma-Aldrich)-coated No. 1.5 coverslips, fixed with 4% paraformaldehyde, permeabilized with 0.1% Triton X-100 for 5 minutes, and then blocked with 5% bovine serum albumin in phosphate-buffered saline. The cells were stained with the indicated primary antibodies and appropriate Alexa fluorophore–conjugated secondary antibodies (Thermo Fischer Scientific) listed in Supplementary Table 3. The resulting samples were mounted with ProLong Gold Antifade (Thermo Fisher Scientific) before microscopic analysis.

To prepare thin-layered samples with suspension cultures (CEM-SS and Human CD4^+^ T cells), 0.25 × 10^6^ cells (400 μL) were put into a cuvette assembled with a poly-L-lysine (Millipore Sigma)-coated No. 1.5 coverslip (Marienfeld Superior) and a cytoclip slide clip, then centrifuged at 1,500 rpm for 4 minutes using Cytospin 3 (Thermo Scientific Shandon). The resulting cells mounted on coverslips were fixed with 4% paraformaldehyde or 1:1 methanol and acetone mixture (for γ-tubulin staining only) and then immunostained as described above.

### Confocal microscopy and three-dimensional structured illumination microscopy (3D-SIM).

Confocal images were acquired under the confocal mode of the Zeiss ELYRA S1 super-resolution microscope (Zeiss) equipped with an Alpha Plan-Apo 63×/1.46 oil objective, 405 nm/488 nm/561 nm/640 nm laser illumination, and standard excitation and emission filter sets. To quantify fluorescence signal intensities, images were acquired under the same laser intensities, converted to the maximum intensity–projected images of multiple z-stacks, and then analyzed using the Zeiss Zen v2.1 software.

For 3D-SIM microscopy, images were acquired by the same ELYRA S1 microscope and then processed using the ZEN black software (Zeiss).

### TEM tomography and segmentation.

#### Sample preparation for electron microscopy.

U2OS cells stably expressing mGFP-Vpr grown on 35 mm gridded glass bottom dishes (MatTek Corporation) were subjected to light microscopy imaging. The resulting cells were fixed with Karnovsky’s fixative, then post-fixed, stained, and resin-embedded as described previously ^81^, except that 2% OsO_4_ and 1% uranyl acetate were used. In addition, a graded series of Polybed resin (EMS) with ethanol was utilized (2:1 ethanol to resin, 1:1 ethanol to resin, and 1:2 ethanol to resin each for 1 hour). Finally, the cells were incubated at room temperature for approximately 48 hours in resin without activator, followed by a 4-hour incubation in resin with activator BDMA at 32 °C. The cells had a final exchange with degassed resin and were allowed to polymerize for 24 hours at 65 °C. The resin was separated from the glass coverslip by heat shock, and any remaining glass particles were removed by hydrofluoric acid treatment and subsequent washing in ddH_2_O. Regions of interest identified by light microscopy imaging were cut out with a jeweler’s saw and glued to resin blocks using super glue. Serial sections were cut *en face* on an ultramicrotome and collected on formvar-coated slot grids. Grids were post-stained with uranyl acetate and lead citrate according to standard protocols, then were carbon coated before being transferred to the TEM for tomography.

#### Tomographic collection and reconstruction.

Areas identified as targets were first exposed to the microscope beam for 10 minutes before tilt series collection to induce resin section collapse (baking) before imaging. Tilt series were collected on a 120 kV Talos L120C TEM (Thermo Fisher Scientific). Images were recorded using a Ceta 16M CCD camera. Tilt series were recorded using SerialEM ^82^ software at a magnification corresponding to a pixel size of 0.966 or 0.595 nm with 1-degree tilt increments over an angular range of ±60° by using a Model 2020 advanced tomography holder (Fischione). Tilt series were reconstructed by using Etomo in the IMOD software package, version 4.11 ^83^, utilizing patch tracking and weighted back-projection with the simultaneous iterative reconstruction-like filter for the final 3D volume. Serial reconstructed volumes were joined using the join serial tomograms module in Etomo. Tomogram movies were created using Quicktime.

#### Segmentation of centrioles.

Before segmentation, tomograms were binned volumetrically by 2 or 4 in IMOD and trimmed to the sub-volume containing centriole clusters. The processed tomograms were loaded into 3D Slicer Version 5.0.2 (https://www.slicer.org) ^84^, and median and Gaussian blur filters were applied. The centrioles and matrix around the centrioles were segmented by using threshold-assisted painting. The resulting 3D models were used to create figures and movies.

### Chromosome spread.

Metaphase chromosome spread was performed as described previously ^85^. In brief, T cells infected with HIV-1 WT or HIV Vpr(1–79) were cultured by plating the cells on an anti-CD3 and anti-CD28 antibody–coated plate for 4 days. Uninfected control cells were simultaneously prepared by following the same procedure without the virus. The resulting cells were treated with 0.1 μg/mL of colcemid (Thermo Fisher Scientific) for 45 minutes, harvested, then resuspended in a prewarmed 75 mM KCl (hypotonic solution) at 37 °C for 8 minutes. The samples were washed three times with freshly prepared fixative (3 methanol:1 glacial acetic acid), mounted on a slide, and stained with Giemsa stain solution (Thermo Fisher Scientific). Images were acquired using the Keyence inverted fluorescence phase contrast microscope BZ-X710 equipped with a 20× objective lens (zoom 3×).

### Immunoprecipitation and immunoblotting.

Immunoprecipitation was carried out as described previously ^86^ in TBSN buffer (20 mM Tris-HCl [pH 8.0], 120 mM NaCl, 0.5% Nonidet P-40, 5 mM EGTA, 1.5 mM EDTA, 2 mM DTT, 20 mM *p*-nitrophenyl phosphate, and protease inhibitor cocktail [Roche]). Immunoblotting was performed using enhanced chemiluminescence reagents (Thermo Fisher Scientific), and signals were captured using a chemiluminescence imager (ChemiDoc^™^ Imaging Systems, Bio-Rad Laboratories). Where indicated, the signal intensities in the immunoblots were quantified using ImageJ (NIH) or Image Lab (Bio-Rad) software. All the antibodies used in this study are listed in Supplementary Table 3.

### ZZ/FLAG-affinity purification and mass spectrometry.

For ZZ-tag affinity purification of Plk4 CPB (581–884) (pKM4285) or its control vector (pKM4283), total cellular lysates prepared from HEK293T cells transfected with the respective construct were subjected to affinity purification with a human IgG (Amersham Biosciences) column. After digestion with AcTEV protease (Invitrogen), proteins were eluted with TBSN buffer for further analysis. For FLAG-affinity purification of Plk4 CTD (581–970) (pKM4591) or its control vector (pKM4067), HEK293T cells that stably expressed them using a lentiviral expression system were subjected to α-FLAG immunoprecipitation. Affinity-purified proteins were separated by 10% sodium dodecyl sulfate-polyacrylamide gel electrophoresis (SDS-PAGE). The protein bands of interest excised from the gel were subjected to in-gel digestion with trypsin (Promega, Madison, WI) followed by nanoLC-tandem mass spectrometry, as described previously ^75^. All the identified peptides are provided in Supplementary Table 4 and some are shown in Fig. 2a and Supplementary Fig. 2a.

### Protein expression and purification.

Purification of CPB (581–808) using the His-MBP-TEV-CPB(581–808) construct (pKM3677) was described previously ^37^. To purify His-CPB (pKM5444), His-MBP-CPB (pKM3686), His-MBP-Plk4 WT (pKM4601), and His-MBP-Plk4 K41M (pKM4602), *E. coli* Rosetta strains (Novagen) expressing the respective constructs were cultured and the proteins were induced with 1 mM Isopropyl β-D-1-thiogalactopyranoside overnight at 16 °C. The resulting cells were lysed in an ice-cold buffer (20 mM Tris-HCl [pH 7.5], 700 mM NaCl, 0.5 mM TCEP), and the lysates were subjected to HisTrap HP column (GE Healthcare) and HiLoad 16/60 Superdex 200 (GE Healthcare) SEC. Purified proteins were stored in the final buffer (20 mM Tris-HCl [pH 7.5], 700 mM NaCl, 0.5 mM TCEP) at −80 °C until use.

VprBP and Vpr were expressed in an improved insect cell line, *Trichoplusia ni* (Tni)-FNL ^87^. To purify the protein, transfected insect cells were cultured in Sf-900 III SFM at 27 °C for 2.5 days, lysed in an ice-cold buffer (20 mM Tris-HCl [pH 7.5], 500 mM NaCl, and 10% [v/v] glycerol), and then subjected to the HisTrap HP column (GE Healthcare). The HisTrap eluate was digested via TEV, mixed with Plk4 CPB, and then subjected to SEC in 20 mM Tris-HCl (pH 7.5), 100 mM NaCl, and 10% (v/v) glycerol.

### Ubiquitination assays.

The CRL4-VprBP-Vpr E3 ligase was assembled by mixing equal molar amounts of separately purified CUL4A-RBX1, DDB1-VprBP (full-length), and Vpr as reported previously ^49,88,89^. E1 (UBA1) and E2 (UBC5B) were purified as previously described ^49^. Typically, E1 (0.8 μM), E2 (5 μM), and appropriate E3 ubiquitin ligase (0.6 μM) were incubated with 2 μM Plk4 or UNG2 and 5 μM ubiquitin in a reaction buffer containing 10 mM Tris-HCl (pH 7.5), 150 mM NaCl, 5% glycerol, 20 units/mL pyrophosphatase, 1 mM Tris(2-carboxyethyl)phosphine, and 5 mM ATP. The reactions were terminated with SDS-PAGE sample loading buffer, and immunoblotting with appropriate antibodies revealed the extent of ubiquitination.

### iSCAMS.

The iSCAMS measurement was carried out using a Refeyn TwoMP mass photometer (Refeyn Ltd.). Cleaned coverslips were assembled into flow chambers. All the buffers used for analysis were filtered through a syringe filter with 0.45-μm pore size (Anotop 10, Whatman). For measurements, all samples were freshly diluted from stock solutions. Sample proteins were diluted to the final concentration of 20 nM in 20 mM HEPES (pH 7.5) buffer containing 200 mM or 400 mM NaCl. The protein solution was loaded into the sample well after finding the focus with the buffer. Obtained data were processed with the DiscoverMP program (Refeyn Ltd.)

### Statistical analysis.

All the experiments were performed at least three times independently. All values are given as mean of n ± s.d. *P* values were calculated by unpaired two-tailed *t*-test from the mean data of each group.

## Data Availability

All the raw data used for quantification and statistical analyses are available from the corresponding author upon request.
